# Biopsy strategies for endoscopic screening of pre-malignant gastric lesions

**DOI:** 10.1038/s41598-019-51487-0

**Published:** 2019-10-17

**Authors:** Meng Zhang, Shan Liu, Yue Hu, Hai-biao Bao, Li-na Meng, Xiao-teng Wang, Yi Xu, Jing Zhao, Bin Lu

**Affiliations:** 1 Department of Gastroenterology, First Affiliated Hospital of Zhejiang Chinese Medical University, Zhejiang, China; 2 Department of Gastroenterology, Wuhan City Jinyintan Hospital, Wuhan, China; 3 Center of Clinical Evaluation, The First Affliated Hospital of Zhejiang Chinese Medical University, Zhejiang, China; 4 Key Laboratory of Digestive Pathophysiology of Zhejiang Province, First Affiliated Hospital of Zhejiang Chinese Medical University, Zhejiang, China

**Keywords:** Gastric cancer, Gastritis

## Abstract

Operative Link on Gastritis Assessment (OLGA) and Operative Link on Gastric Intestinal Metaplasia Assessment (OLGIM) were adopted to evaluate gastric risk stratification in five biopsy samples. This study aimed to evaluate the degree of gastric atrophy (GA) and intestinal metaplasia (IM) in five locations to detect a more representative biopsy sample in gastric cancer (GC) screening. Our study enrolled 368 patients and 5 biopsy pieces were acquired from them. Gastric risk stratification was calculated by OLGA and OLGIM staging system. The results revealed that the IM score in the incisura angularis was higher than that in the larger and lesser curvature of corpus mucosa (*p* = 0.037 and *p* = 0.030, respectively) and the IM score in the lesser curvature of antrum mucosa was higher than that in the incisura angularis mucosa (*p* = 0.018). IM is more frequently observed in the angulus region than in the lesser curvature of corpus in the mild degree (*p* = 0.004) and mild IM lesions in the lesser curvature of antrum were more frequently observed than in the incisura angularis mucosa (*p* = 0.004), Four biopsy pieces protocol (larger curvature and lesser curvature of the antrum, lesser curvature of the corpus and angulus) demonstrated accurate consistency (97.83% and 98.37%, respectively) with a Kendall’s tau-b of higher than 0.990, along with low misdiagnosis rates of OLGA and OLGIM (III + IV) (9.76% and 5.00%, respectively). Three biopsy pieces protocol (lesser curvature of the antrum and corpus, angulus biopsy) in OLGA and OLGIM staging system was close to the standard protocol (five biopsy specimens) with a consistency of 94.84% and 94.29% and has a Kendall’s tau-b higher than 0.950 and diagnostic omission rates of 9.76% and 5.00%, respectively, which was exactly the same with the four biopsy pieces protocol. Furthermore, it had the second-highest Youden index (0.902 and 0.950, respectively) and area under the ROC curve (0.992 and 0.996, respectively) for the screening of high-risk GC by OLGA and OLGIM stages. Thus, we recommended the angulus and the lesser curvature of antrum as a conventional biopsy and three biopsy pieces for further GC risk screening.

## Introduction

GA and IM are detectable precursor lesions in most of the gastric carcinomas. Early endoscopic examination is of paramount importance in the early detection and treatment of advanced precursor lesions and cancer. The annual incidence of GC is quite low in western populations, for GA was 0.1% and for IM was 0.25%^1,2^. Though only few patients with GA or IM ultimately developed into gastric cancer, it is still necessary for endoscopic surveillance for the early detection of GC. Japan is a country with high incidence rates of GC among men and women, mass nationwide GC risk screening programs generated a greater detection rate of early gastric carcinoma, which eventually reduced the mortality rates^3,4^. This strategy is also applied for China as the GC being the second highest type of carcinoma^5^.

There were several screening tools put forward for GC surveillance but the potentially useful test is endoscopy. Some controversies still remain in the endoscopic surveillance of premalignant lesions. Histological assessment and biopsy sampling protocol has been standardized according to the updated Sydney system. The recommended five gastric biopsies that are widely applied in the staging system for GC risk stratification. The operative links on gastric assessment (OLGA and OLGIM) that addresses the grade and extension for GA and IM. It is deemed that OLGA stages 0-II are associated with low risk, OLGA stages III and IV with high risk^2,6,7^. The long-term (12 years) follow-up demonstrated that a high-stage gastritis at enrollment significantly predicted neoplasia at the end of follow-up^8^, while a gross of the high-risk developed a significant progression in 3 years^9^. For more than a decade, the recommended five-biopsy sample protocol remained unchanged. While some believe that the GA risk screening requires consideration of cost, suffering of patients as well as rational utilization of medical resources, so adopting the ‘five biopsy’ protocol may presumably be less appropriate in large-scale population for gastric cancer risk screening.

In this study, we evaluated the OLGA and OLGIM staging in a standardized five-biopsy protocol through screening of patients who underwent endoscopy with abdominal pain and discomfort. In order to identify a more optimal biopsy strategy with high consistency with the standardized five biopsy protocol in OLGA and OLGIM staging and less number of biopsies during cancer risk assessment, we re-evaluated OLGA and OLGIM staging by adopting different biopsy combinations (evaluated the appropriate biopsy locations and number of biopsies).

## Material and Methods

### Study design and participants

Three hundred and eighty nine patients with an age ranging from 18 to 82 years and who underwent upper endoscopy for epigastric discomfort and other clinical reasons in The First Affiliated Hospital of Zhejiang Chinese Medical University were enrolled into the study prospectively from August 2015 to August 2016.

The study protocol was conformed to the ethical guidelines of the 1975 Declaration of Helsinki and approved in August 2015 by the ethics Committee of The First Affiliated Hospital of Zhejiang Chinese Medical University (2015-Q-004-02). All patients were written informed consent prior to participation in the study. Exclusion criteria of the patients were as follows: cancer, postgastrectomy, oral antibiotics or anticoagulation for the last half month, some severe systemic diseases (e.g. serious cardiac dysfunction, severe liver malfunction, renal failure or severe mental disorder). Conventional endoscope (GIF 260 Olympus Medical system, Tokyo, Japan) and disposable endoscopic biopsy forceps (Micro-Tech (Nanjing) CO., Ltd) were employed.

Totally five biopsy pieces (two from antrum, namely lesser and larger curvature of the antrum at 3 cm distance from the pylorus, two from corpus with larger and lesser curvature of the middle corpus and one from incisura angularis) were acquired in strict accordance with the updated Sydney system^2^. The biopsies were immediately fixed in 10% formaldehyde solution and embedded in paraffin for further histological assessment and detection of Helicobacter pylori (*H. pylori*) status.

### Histological assessment and *H. pylori* status

All biopsy specimens were tested by experienced pathologists. Pathologists have access to only the basic demographic information and a short depiction of the endoscopic characteristics of each patient. Sections were conducted by H&E staining, periodic acid-Schiff staining (PAS), and the modified Giemsa staining method for determination of *H. pylori* infection. Positive findings in histology were regarded as having a positive *H. pylori* status and vice verse. The score of GA and IM were conducted on a visual analogue scale (VAS) based on the updated Sydney system (0 = none, 1 = mild, 2 = moderate and 3 = severe)^7^.

### Statistical analysis

Continuous variables are expressed as mean ± SD, while discrete variables are expressed as n (%). Chi-square test was used to compare categorical variables such as baseline and clinicopathologic characteristics between chronic superficial gastritis group and chronic atrophic gastritis subjects. Student’s t-test was used to compare the mean age differences. Kendall’s tau test was applied to compare the degree of correspondence between OLGA and OLGIM stages. The consistency of different number of biopsy specimen combinations (two pieces, three pieces, and four pieces, respectively) was compared with the standard 5 biopsy specimens based on OLGA and OLGIM stages by Kendall’s tau test. The biopsy locations and number of biopsies for screening of high-risk GC in OLGA and OLGIM stages was further analysised by ROC analysis. The Kruskal-Wallis H-test is a nonparametric test and with paired comparison the total score of GA and IM of the five-biopsy samples was analyzed. Mann-Whitney U test with bonferroni correction was applied to evaluate the three different degrees (mild, moderate, and severe) of GA and IM of the five-biopsy samples, respectively. SPSS version 22.0 statistical software (SPSS Inc., Chicago, IL, United States) was used for all statistical analyses. All *p*-values reported are two-tailed, and *p*-values of <0.05 were considered to be statistically significant different, while a *p*-value of <0.005 was considered to be statistically significant difference for Mann-Whitney U test with bonferroni correction.

### Compliance with ethical standards

This study was approved by local Ethical Committee of the First Affiliated Hospital of Zhejiang Chinese Medical University.

## Results

### Demographic information and *H. pylori* status

Altogether 389 patients participated in this study, the pathologic result showed that 5 patients had gastric signet-ring cell carcinoma, 12 had gastric adenocarcinoma and 3 had gastric non-hodgkin’s lymphoma, and 1 had neuroendocrine neoplasm, which were excluded in this study. Ultimately, 368 patients (161 male, 207 female; age range 20–82years, 52.90 ± 13.29) were enrolled, among which 156 had chronic superficial gastritis and 212 had chronic atrophic gastritis. H. pylori showed a positive result in 99 cases (26.9%). There were no significant differences in sex, family history of gastric carcinoma, smoking, alcohol drinking and H. pylori infection between chronic superficial gastritis group and chronic atrophic gastritis group (all p < 0.05), while the mean age of patients in chronic atrophic gastritis group (56.52 ± 12.09) was higher than those patients in chronic superficial gastritis group (47.99 ± 13.30), (t = 6.413, p < 0.001). Demographic information and results of H. pylori detection in the study participants are summarized in Table 1.

**Table 1:** Demographic information and results of Helicobacter pylori detection in the study patients.

Histological diagnosis	N	M:F	Age (yrs, mean ± SD)	FHGA (n, %)	Smoking (n, %)	Alcohol (n, %)	Histology (positive subjects/total subjects, n, %)
Non-atrophic gastric	156	62:94	47.99 ± 13.30	2 (1.3)	16 (10.3)	45 (28.8)	38/156 (24.4)
Atrophic gastric	212	99:113	56.52 ± 12.09	4 (1.9)	23 (10.8)	79 (37.3)	61/212 (28.8)
Total	368	161:207	52.90 ± 13.29	6 (1.6)	39 (10.6)	124 (33.7)	99/368 (26.9)

N, number; F, female; M, male; FHGA, family history of gastric carcinoma.

### The degree of atrophy and IM in five biopsy specimens

The number of patients by grading of GA and IM are displayed in Table 2. Mild atrophic changes were most frequently observed in the lesser curvature of the antrum accounting for 33.03%(109/330) of those with whole mild atrophic changes, while moderate and severe atrophic changes with the highest frequency of 32.91%(52/158) and 40.00%(18/45) in incisura angularis were observed. The degree of GA of the five locations showed significant difference (*H* = 11.281, *p* = 0.024), while there were no significant differences between larger curvature of the corpus, lesser curvature of the corpus, incisura angularis, larger curvature of the antrum, lesser curvature of the antrum with respect to total GA score and three different degrees of GA lesions.

**Table 2:** Number of patients by grade of atrophy and intestinal metaplasia in the larger curvature of corpus, in the lesser curvature of corpus, in the incisura angularis, in the larger curvature of antrum and in the lesser curvature of antrum.

Preneoplastic condition	Larger curvature of the corpus	Lesser curvature of the corpus	Incisura angularis	Larger curvature of the antrum	Lesser curvature of the antrum
Atrophic gastritis					
Mild (1)	28	54	73	66	109
Moderate (2)	10	17	52	31	48
Severe (3)	3	6	18	5	13
Intestinal metaplasia					
Mild (1)	29	53	68^◆#^	66	111
Moderate (2)	6	16	47	31	43
Severe (3)	3	6	20	5	12

Mann–Whitney U test with bonferroni correction.

^#^Incisura angularis vs lesser curvature of the corpus, *p* < 0.005.

^◆^Incisura angularis vs Lesser curvature of the antrum, *p* < 0.005.

Of the whole mild IM changes, the lesser curvature of the antrum mucosa had the highest incidence rates of 33.94%(111/327) of the whole mild IM changes, wihle the incisura angularis had the highest frequency of 32.87%(47/143) and 40.00%(20/46) in moderate and severe IM alterations. The score of IM lesions of the five locations showed significant differences (*H* = 16.092, *p* = 0.003).The IM score in the incisura angularis was higher than that in the larger and lesser curvature of corpus mucosa (*z* = 2.899, *p* = 0.037 and *z* = 2.964, *p* = 0.030, respectively) and IM score in the lesser curvature of antrum mucosa was higher than in the incisura angularis (*z* = 3.125, *p* = 0.018).What’s more, the mild IM changes(with score 1) were more frequently observed in the incisura angularis than in the lesser curvature of corpus mucosa (*z* = 2.845, *p* = 0.004, Table 2) and mild IM changes in the lesser curvature of antrum were more frequently observed than in the incisura angularis mucosa (z = 2.895, *p* = 0.004, Table 2).

**Figure 1:**
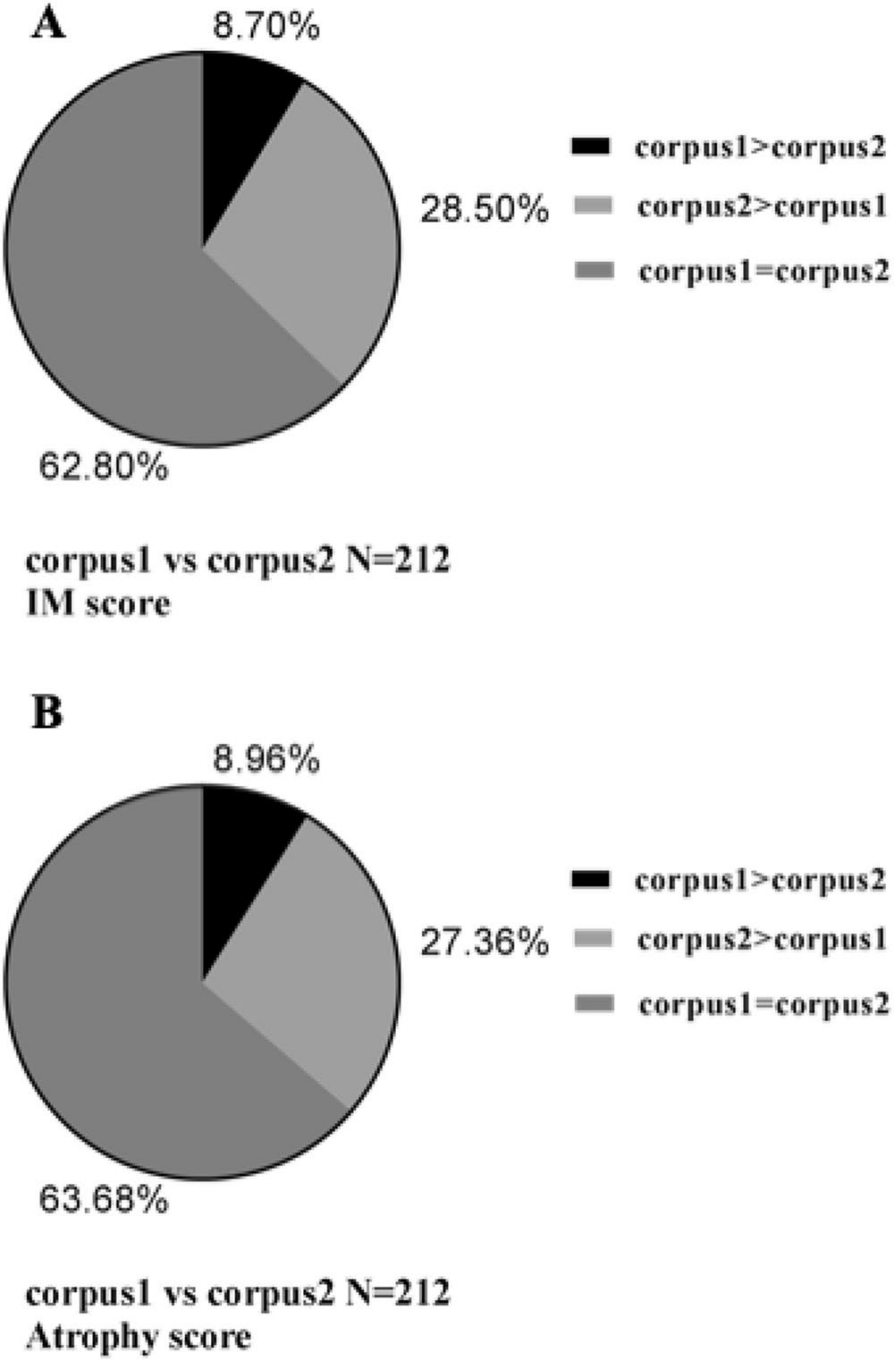
The IM and atrophy score distribution of the lesser curvature and larger curvature of corpus. (**A**) The IM score. (**B**) The atrophy score. Corpus 1, the larger curvature of corpus; corpus 2, the lesser curvature of corpus; IM, intestinal metaplasia.

**Figure 2:**
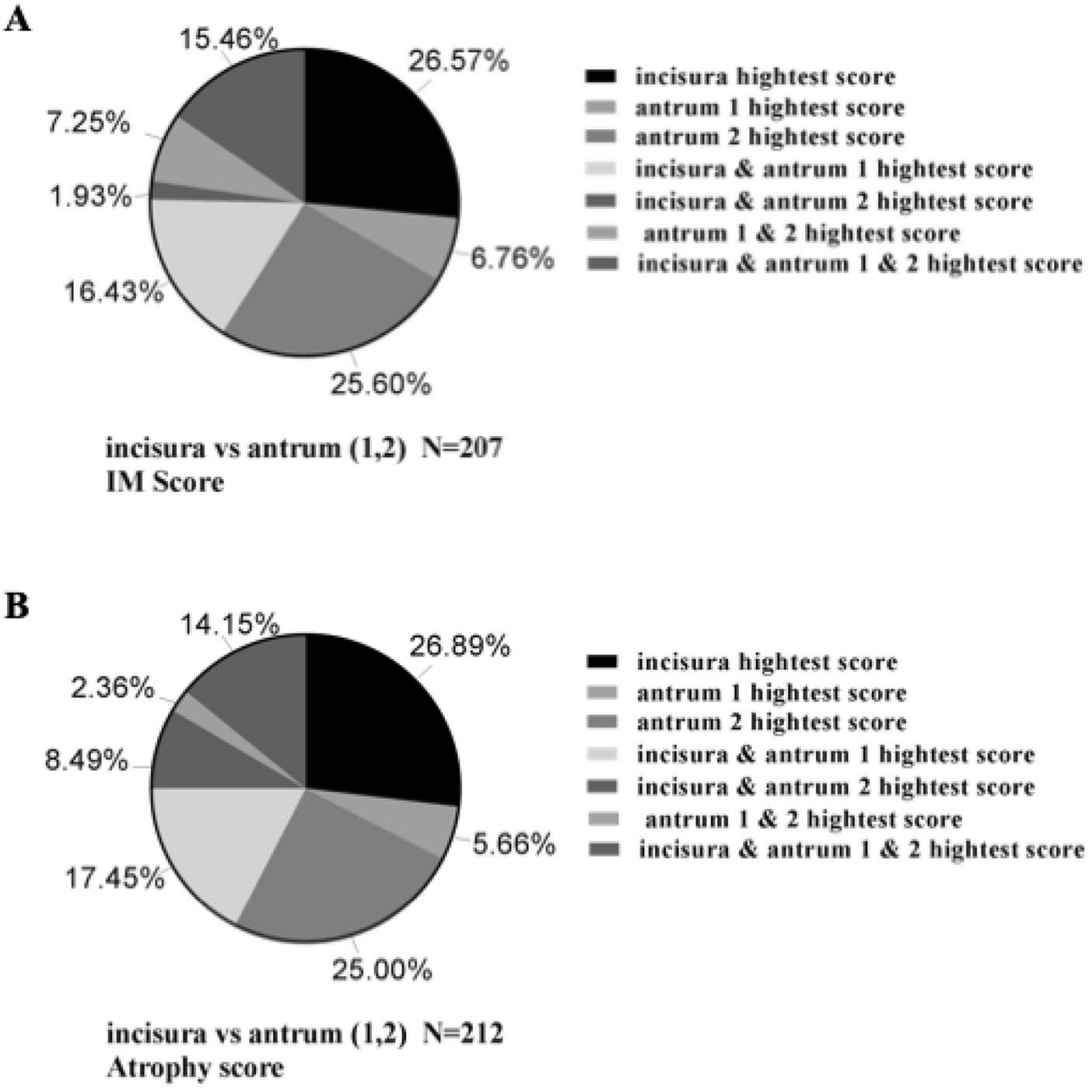
The IM and atrophy score distribution of the lesser curvature and larger curvature of antrum and incisura angularis. (**A**) The IM score. (**B**) The atrophy score. Antrum 1, the larger curvature of antrum; antrum 2, the lesser curvature of corpus; IM, intestinal metaplasia.

Lesser curvature was only adopted, which accounted for 91.04% and 91.30% of the total atrophy and IM in the corpus, respectively, while the larger curvature alone accounted for 71.50% and 72.64%. The atrophy and IM score distribution of the lesser curvature and larger curvature of the corpus are shown in Fig. 1. In the antrum, the rate was slightly lower as three pieces are obtained. The lesser curvature alone accounted for 65.09% and 64.73% of general atrophy and IM, separately. However, the larger curvature alone occupied 30.66% and 31.40%. The atrophy and IM score distribution of the lesser curvature and the larger curvature of the antrum and the incisura angularis are shown in Fig. 2.

### Risk stratification of OLGA/OLGIM and biopsy protocol

The distribution of patients in OLGA and OLGIM was presented in Table 3. The OLGA and OLGIM stages distribution showed a high accordance of 96.74% (Kendall’s tau-b = 0.968, *p* < 0.001). The OLGA and OLGIM stages were re-evaluated according to different biopsy specimens and compared with the updated Sydney system^7^ (five biopsy specimens) in order to explore a more simpler and good consistency biopsy protocol. Approximately half of the patients in OLGA and OLGIM have scored 0 (156 and 161, respectively). Patients’ staging was based on the same histological criteria of different biopsy specimens both in OLGA and OLGIM, and was shown in Table 4 and 5, respectively. Compared with the standard protocol (five biopsy specimens), the consistency of four biopsy samples (contains 5 different combinations) and three biopsy samples (contains 9 different combinations) were quite high, ranging from 86.14% to 97.83% and 65.76% to 94.84% in OLGA staging systems (Kendall’s tau-b ranged from 0.874 to 0.992 and from 0.719 to 0.964, respectively), while in OLGIM staging systems they ranged from 86.41% to 98.37% and ranged from 66.30% to 94.29% (Kendall’s tau-b ranged from 0.876 to 0.990 and ranged from 0.721 to 0.952, respectively). But the consistency of the two-biopsy samples (containing 6 different combinations) were quite low, which ranged from 60.33%-79.62% in OLGA staging systems (Kendall’s tau-b ranged from 0.629 to 0.828) and 61.41% to 79.89% in OLGIM staging systems (Kendall’s tau-b ranged from 0.639 to 0.825). In comparison with the standard protocol (five biopsy specimens), four biopsy samples (lesser and larger curvature of the antrum, lesser and larger curvature from corpus), the incisura angularis were not taken into account, manifested the lowest consistency of 86.14% in OLGA staging system and 86.41% in OLGIM staging system (Kendall’s tau-b = 0.874 and 0.876, respectively). Nevertheless, the four-biopsy samples without of the larger curvature of the antrum showed the highest consistency of 97.83% and 98.37% in OLGA and OLGIM staging systems (Kendall’s tau-b = 0.992 and 0.990, respectively). Of note, a rather high accordance of 94.84% in OLGA (Kendall’s tau-b = 0.964) and 94.29% in OLGIM (Kendall’s tau-b = 0.952) were unfolded in three biopsy samples (lesser curvature of the antrum and corpus, angulus biopsy), which was even better than four biopsy sample protocols (the standard protocol excluding the lesser curvature of antrum or incisura angularis). However, when two biopsy protocols were applied, the peak consistency of 79.62% and 79.89% was observed, which was a fairly low accordant rate was found in the lesser curvature of the antrum and the corpus protocoll in OLGA and OLGIM staging systems (Kendall’s tau-b = 0.828 and 0.825, respectively).

**Table 3:** Number of patients according to OLGA and OLGIM stage.

		Stage 0	Stage I	Stage II	Stage III	Stage IV	Total
OLGIM							
OLGA	Stage 0	156	0	0	0	0	156
	Stage I	3	106	0	0	0	109
	Stage II	2	5	55	0	0	62
	Stage III	0	0	1	26	1	28
	Stage IV	0	0	0	0	13	13
	Total	161	111	56	26	14	368

OLGA, Operative Linkon Gastritis Assessment; OLGIM, Operative Link on Gastric Intestinal Metaplasia Assessment.

**Table 4:** The consistency of different number of biopsy specimen protocols (two pieces, three pieces, four pieces, respectively) compared with the standard 5 biopsy specimens protocol based on OLGA stage.

	Stage0	StageI	StageII	StageIII	StageIV	Total	Consistency (%)	Kendall’s tau-b	*P* value
OLGA									
Five biopsy pieces (C1C2A3A1A2)	156	109	62	28	13	368	100	1.000	—
four biopsy pieces (C1C2A3A1)	192	86	55	22	13	368	86.68	0.874	<0.001
four biopsy pieces (C1C2A3A2)	161	111	56	27	13	368	96.74	0.971	<0.001
four biopsy pieces (C1C2A1A2)	177	111	50	23	7	368	86.14	0.881	<0.001
four biopsy pieces									
(C2A3 A1A2)	156	111	64	26	11	368	97.83	0.992	<0.001
four biopsy pieces									
(C1A3 A1A2)	159	106	71	28	4	368	94.29	0.982	<0.001
three biopsy pieces (C1C2A3)	209	83	46	20	10	368	78.26	0.804	<0.001
three biopsy pieces (C1C2A1)	230	83	40	9	6	368	65.76	0.719	<0.001
three biopsy pieces (C1C2A2)	186	112	41	22	7	368	82.07	0.839	<0.001
three biopsy pieces (C1A3A1)	200	78	63	23	4	368	79.89	0.850	<0.001
three biopsy pieces (C1A1A2)	182	112	52	19	3	368	82.61	0.844	<0.001
three biopsy pieces (C1A3A2)	164	108	64	28	4	368	91.30	0.954	<0.001
three biopsy pieces (C2A3A1)	192	89	54	23	10	368	84.78	0.866	<0.001
three biopsy pieces (C2A1A2)	180	110	52	20	6	368	83.70	0.870	<0.001
three biopsy pieces (C2A3A2)	161	113	57	26	11	368	94.84	0.964	<0.001
two biopsy pieces (C1A3)	220	74	51	19	4	368	73.37	0.761	<0.001
two biopsy pieces (C1A1)	254	71	33	7	3	368	60.33	0.629	<0.001
two biopsy pieces (C1A2)	192	112	43	18	3	368	78.53	0.800	<0.001
two biopsy pieces (C2A3)	211	83	47	18	9	368	77.45	0.788	<0.001
two biopsy pieces (C2A1)	239	80	35	10	4	368	63.32	0.682	<0.001
two biopsy pieces (C2A2)	189	112	41	20	6	368	79.62	0.828	<0.001

Abbreviations: C1, larger curvature of the corpus; C2, lesser curvature of the corpus; A3, incisura angularis; A1, larger curvature of the antrum; A2, lesser curvature of the antrum.

**Table 5:** The consistency of different number of biopsy specimen protocols (two pieces, three pieces, four pieces, respectively) compared with the standard 5 biopsy specimens protocol based on OLGIM stage.

	Stage0	StageI	StageII	StageIII	StageIV	Total	Consistency (%)	Kendall’s tau-b	*P* value
**OLGIM**									
Five biopsy pieces (C1C2A3A1A2)	161	111	56	26	14	368	100-	1.000	—
Four biopsy pieces (C1C2A3A1)	197	85	49	22	14	368	86.96	0.876	<0.001
four biopsy pieces (C1C2A3A2)	167	113	48	26	14	368	96.47	0.967	<0.001
four biopsy pieces (C1C2A1A2)	180	113	47	21	7	368	86.41	0.892	<0.001
four biopsy pieces (C2A3 A1A2)	163	110	57	25	13	368	98.37	0.990	<0.001
four biopsy pieces (C1A3 A1A2)	164	108	66	25	5	368	94.02	0.981	<0.001
three biopsy pieces (C1C2A3)	214	81	42	19	12	368	79.62	0.810	<0.001
three biopsy pieces (C1C2A1)	235	81	37	10	5	368	66.30	0.721	<0.001
three biopsy pieces (C1C2A2)	191	114	36	20	7	368	81.52	0.839	<0.001
three biopsy pieces (C1A3A1)	205	78	59	21	5	368	79.89	0.854	<0.001
three biopsy pieces (C1A1A2)	184	116	50	15	3	368	82.07	0.860	<0.001
three biopsy pieces (C1A3A2)	170	110	58	25	5	368	90.49	0.950	<0.001
three biopsy pieces (C2A3A1)	199	86	48	24	11	368	85.33	0.870	<0.001
three biopsy pieces (C2A1A2)	183	111	50	18	6	368	84.51	0.881	<0.001
three biopsy pieces (C2A3A2)	170	111	49	27	11	368	94.29	0.952	<0.001
two biopsy pieces (C1A3)	225	74	47	17	5	368	73.37	0.764	<0.001
two biopsy pieces (C1A1)	256	72	31	6	3	368	61.41	0.639	<0.001
two biopsy pieces (C1A2)	195	116	40	14	3	368	77.72	0.809	<0.001
two biopsy pieces (C2A3)	218	79	42	20	9	368	77.72	0.791	<0.001
two biopsy pieces (C2A1)	241	79	34	10	4	368	64.67	0.699	<0.001
two biopsy pieces (C2A2)	194	113	37	18	6	368	79.89	0.825	<0.001

Abbreviations: C1, larger curvature of the corpus; C2, lesser curvature of the corpus; A3, incisura angularis; A1, larger curvature of the antrum; A2, lesser curvature of the antrum.

In the standard protocol, the number of patients classified in high risk OLGA stages (III + IV) was 41. However, the number in high risk OLGA stages showed no significant changes in the four biopsy sample protocol (the standard protocol excluded the lesser curvature of corpus) and in the three biopsy sample protocol (lesser curvature of the antrum and corpus, angulus biopsy), (37 VS 41, 37VS 41, respectively). However, four biopsy sample protocol (thestandard protocol without incisura angularis) generated a marked decrease in OLGA (III + IV) (30 VS 41), which was approximately to that of the two biopsy sample protocols (lesser curvature of the antrum and the corpus protocol, lesser curvature of the corpus and the incisura angularis protocol) (26 VS 41, 27 VS 41 respectively). Similar results were obtained in OLGIM staging system. In the standard protocol, the number of patients classified in high-risk OLGIM stages (III + IV) was 40. The number in high risk OLGA stages showed similar results in four-biopsy sample protocol (the standard protocol with the exclusion of the lesser curvature of corpus) and three-biopsy sample protocol (lesser curvature of the antrum and corpus, and angulus biopsy), (38 VS 40, 38VS 40, respectively). However, four biopsy sample protocol (standard protocol without the incisura angularis) generated a marked decrease in OLGA (III + IV) (28 VS 40), which was even less than the two biopsy sample protocols (lesser curvature of the antrum and corpus protocol) (29 VS 40).

### The ROC curve of the biopsy locations and number of biopsies for the endoscopic screening of high-risk GC by OLGA and OLGIM stage systems

**Figure 3:**
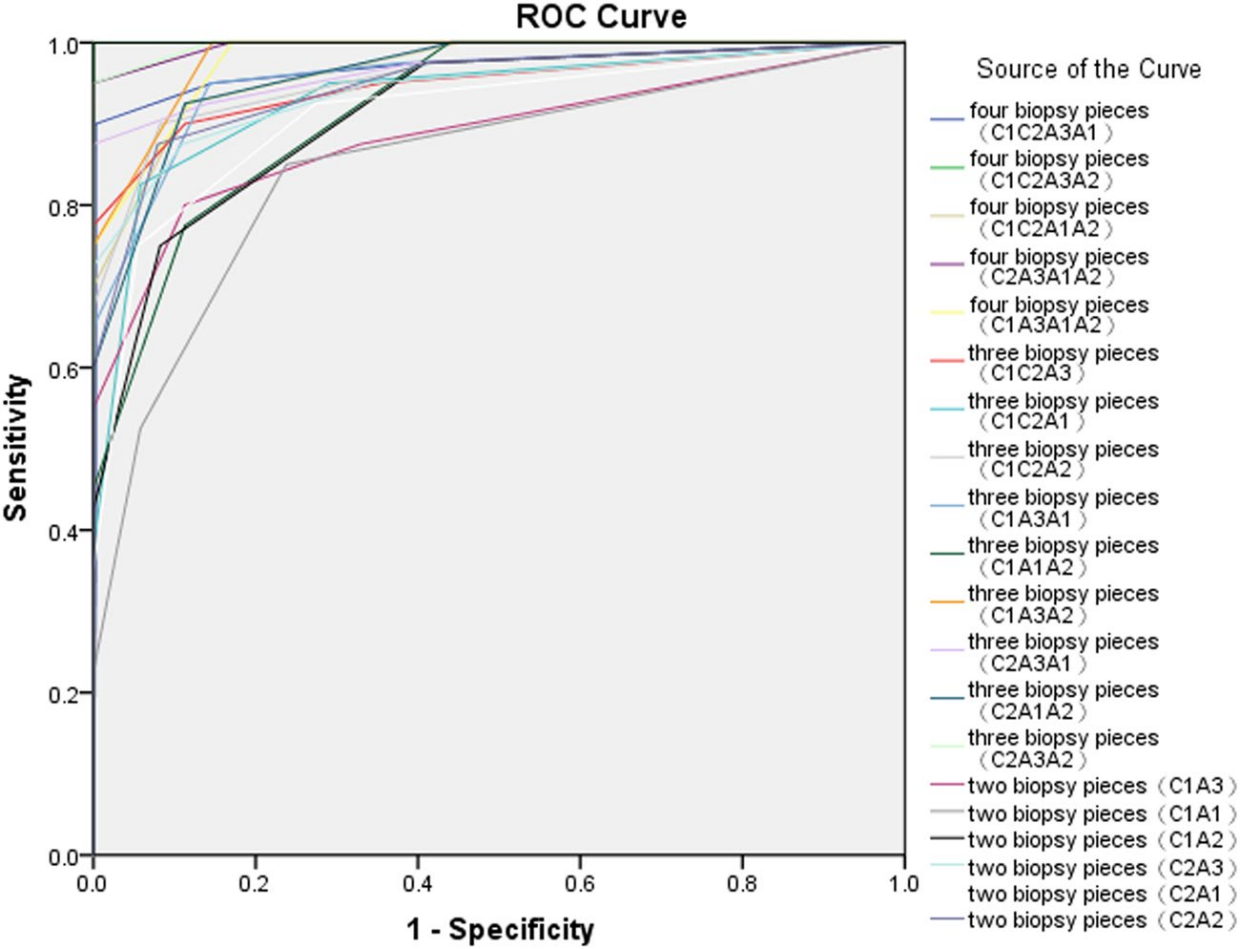
The ROC curve of the biopsy locations and number of biopsies for endoscopic screening of high risk of GC by OLGA stage. C1, larger curvature of the corpus; C2, lesser curvature of the corpus; A3, incisura angularis; A1, larger curvature of the antrum; A2, lesser curvature of the antrum.

**Figure 4:**
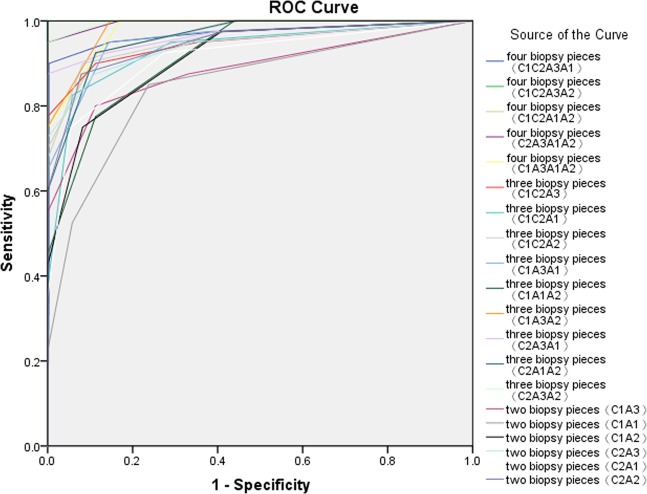
The ROC curve of the biopsy locations and number of biopsies for endoscopic screening of high risk of GC by OLGIM stage. C1, larger curvature of the corpus; C2, lesser curvature of the corpus; A3, incisura angularis; A1, larger curvature of the antrum; A2, lesser curvature of the antrum.

The ROC curve of the biopsy locations and number of biopsies for the endoscopic screening of high-risk GC by OLGA and OLGIM stages is shown in Figs 3 and 4, respectively. The results of ROC analysis showed that four biopsy pieces (the standard protocol with the exclusion of the larger curvature of antrum) had the highest Youden index of 0.976 and area under the ROC curve of 0.998 for the screening of high-risk GC (OLGA stages III–IV). A combination of three biopsy pieces (lesser curvature of the antrum and corpus, and angulus biopsy) had the second-highest Youden index of 0.902 and area under the ROC curve of 0.992. The combination of two biopsy pieces (lesser curvature of the antrum and corpus) had a higher Youden index of 0.838 and area under the ROC curve of 0.968 compared with the combinations of other two-biopsy pieces. The ROC curve of the biopsy locations and number of biopsies for the endoscopic screening of high-risk GC (OLGIM stages III–IV) revealed some similar results. The results indicated that four biopsy pieces (the standard protocol with the exclusion of the larger curvature of antrum) had the highest Youden index of 1.0 and area under the ROC curve of 1.0, consistent with the results on the use of five biopsy pieces for the endoscopic screening of high-risk GC (OLGIM stages III–IV). Four biopsy pieces (the standard protocol with the exclusion of the larger curvature of corpus) and a combination of three biopsy pieces (lesser curvature of the antrum and corpus, and angulus biopsy) had the second-highest Youden index of 0.950 and area under the ROC curve of 0.996. The combination of two biopsy pieces (lesser curvature of the antrum and corpus) had a higher Youden index of 0.796 and area under the ROC curve of 0.947 compared with the combination of other two-biopsy pieces.

## Discussion

The pre-malignant lesions (GA and IM) of gastric mucosa demonstrated a higher prevalence rates in our study (156/368, 42.39% and 161/368, 43.75%) when compared to other the recent reports conducted in other European countries^1,10,11^. While a small proportion (41/368, 11.14% and 40/368, 10.87%) of severe atrophy and IM with heightened risk of development to GC was accounted, and was in accordance with the epidemiological data of Germany^12^. The mean age of chronic atrophic gastritis group was higher than that of the chronic superficial gastritis group. Previous study have confirmed that old age was an independent risk factors for patients with chronic atrophic gastritis and GC and early GC surveillance for individuals aged 40 years and older has already been introduced in China^13–15^.

Incisura angularis is the main region for the early-onset of atrophic-metaplastic transformation^16–19^. Our study found that atrophic and IM changes were more frequently observed in the angularis region than in the lesser and larger curvature of corpus mucosa, especially mild degree (score 1). Thus a biopsy obtained from this location might assist in increasing the detection of IM alterations. The incisura angularis plays a significant role in OLGA and OLGIM staging for assessing GC. The gastritis stage was significantly decreased with a correspondence rate of 86.40% and 86.41% when the OLGA and OLGIM staging systems were employed without considering incisura angularis. Furthermore, high-risk OLGA and OLGIM gastritis stages (III + IV) were decreased by 29.27% (12/41) and 30.00% (12/40), respectively. These findings revealed that significance for the clinical biopsy is imperative to pick up the most appropriate piece that approximated to the true circumstance of the stomach.

Our study also demonstrated that the IM lesions in the lesser curvature of the antrum had the higher occurrence rate than in incisura angulari with the mild degree (score 1). Removal of the lesser curvature of the antrum showed significant downgrading of the gastritis stage with a correspondence rate of 86.68% and 86.96% when OLGA and OLGIM staging systems were employed. This demonstrated that the lesser curvature of the antrum should not be excluded from the analysis. Of note, for a fairly accurate gastritis OLGA and OLGIM staging as well as GC predictionand, it seems that, the incisura angularis is also considered as an indispensable one. If the incisura angularis was added, the overall diagnostic rate was enhanced to 27.36% in atrophy and 28.50% in IM.

Different combination modes of biopsy pieces (at least one piece in the antrum and corpus respectively) using OLGA and OLGIM staging systems were screened to choose the most suitable one over the standard five biopsy pieces to guide clinical operation. For this, the four biopsy pieces protocol (larger curvature and lesser curvature of the antrum, lesser curvature of the corpus and angulus) with high consistency (97.83% and 98.37%, respectively) and a Kendall’s tau-b of higher than 0.990 and rather low diagnostic omission rates (9.76% and 5.00%) in high risk OLGA and OLGIM gastritis stages (III + IV) were discovered. While the result of three biopsy pieces protocol (lesser curvature of the antrum and corpus, angulus biopsy) in OLGA and OLGIM staging systems was close to that of the standard protocol (five biopsy specimens) with a consistency of 94.84% and 94.29% with a Kendall’s tau-b of higher than 0.950 and diagnostic omission rates of 9.76% and 5.00%, this was similar with that of the four biopsy pieces protocol (larger curvature and lesser curvature of the antrum, lesser curvature of the corpus and angulus). The results of ROC curve of the biopsy locations and number of biopsies for the endoscopic screening of high-risk GC by OLGA and OLGIM stage systems showed that the combination of four biopsy pieces (the standard protocol with the exclusion of the lesser curvature of antrum) was the most recommended choice, followed by the combination of three biopsy pieces (lesser curvature of the antrum and corpus, angulus biopsy). The combination of two biopsy pieces was not recommended owing to a low Youden index. The optimized recommendation results of Kendall’s tau-b test and ROC analysis were different. However, the results of the second recommended biopsy combination (lesser curvature of the antrum and corpus, angulus biopsy) showed good agreement. The optimized recommendation of the combination of four pieces (the standard protocol with the exclusion of the larger curvature of antrum) in endoscopic screening of high risk of GC and four pieces protocol (the standard protocol with the exclusion of the larger curvature of corpus) with the highest consistency compared with the standard five biopsy specimens in OLGA and OLGIM staging contained the contained the combination(lesser curvature of the antrum and corpus, angulus biopsy). Thus, in order to be less cost and less painful for patients and more convenient clinical application, at least three biopsy pieces were needed.

In conclusion, the incisura angularis underwent more mild IM alteratons than the lesser curvature of corpus. Thus, the incisura angularis biopsy piece should be routinely picked up in clinical practice, particularly for IM. Furthermore, the lesser curvature of antrum underwent more IM changes than the incisura angularis in the mild degree, further supporting that the lesser curvature of antrum should also routinely be picked up in the biopsy sampling protocol. For a more valid, uncomplicated and affordable prediction of gastritis using OLGA and OLGIM staging systems and GC risk stratification, three-biopsy pieces (lesser curvature of the antrum and corpus, angulus) with a rather high consistency when compared with the standard five-biopsy specimens protocol are recommended.

## Supplementary information

Supplemetary materials
